# Performing in the heat: a new practical midcooling method

**DOI:** 10.1186/2046-7648-4-S1-A130

**Published:** 2015-09-14

**Authors:** Athanasios Zavvos, Panagiotis Gkrilias, Niki Manolaki, Evgenia Cherouveim, Maria Koskolou, Nikolaos Geladas

**Affiliations:** 1 Department of Sports Medicine and Biology of Exercise, Faculty of Physical Education, and Sport Science, University of Athens, Greece; 2 Technological Educational Institute (TEI) of Western Greece, Department of Physical Therapy, Aigio Achaias, Greece

## Introduction

Pre-cooling aims to decrease body core temperature prior to [1] and decelerate its rise during exercise preventing excessive hyperthermia [2]. Exercise time to exhaustion in a hot environment is inversely related to the initial body temperature and directly affected by the rate of heat storage [3]. Despite the fact that all ball games involve intermittent activity with at least one long brake among periods, the majority of existing pre-cooling methods are applied before event initiation and use aggressive techniques (cold-water immersion, ice cubes, ice vests). This practice is cumbersome and may initially induce hypothermia, ensuing thermo genesis and discomfort. The purpose of this study was to investigate the effect of a new, practical method for cooling the body during the break (mid-cooling) of a prolonged, high-intensity intermittent exercise in the heat.

## Methods

Eight healthy subjects (22 (1.7) yrs) performed, in 31 °C, two experimental conditions: mid-cooling (Mid) and (Con), a 46-min intermittent exercise protocol consisting of multiple 2-min bouts (5 sec sprinting on a cycle ergometer against a resistance equal to 7.5% of the subject's body weight, 105 sec energetic rehabilitation at 35% VO_2max_, 10sec of passive recovery) [4]. A 15min break in a thermo-neutral environment (26 (0.58) °C, 50% (0.5) % rh) followed, with subjects remaining idle; in Mid, they were covered with a bathrobe (body) and a towel (feet) both garments previously soaked into water of 17 (0.12) °C. Upon completion of the 15min break, another intermittent exercise protocol, similar to the first, was performed until rectal temperature (Tre) approached 39°C.

## Results

No differences were observed between conditions in the initial 46 min of exercise. Τhe rate of rectal temperature drop (ΔTre) during the break was higher in Mid (-0.15 (0.02) °C) than in Con (-0.05 (0.02) °C) (p = 0.03), whereas the rate of change in skin temperature (ΔTskin) and heart rate (HR) were similar in the two conditions. Upon completion of the second exercise period, the rise of Tre was less profound (p = 0.05) and HR tended to be lower (p = 0.12) in Mid (0.42 (0.07) °C; 139 (1.57) beats.min^-1^) than in Con (0.61 (0.11) °C; 153 (1.20) beats.min^-1^), whereas ΔTskin did not differ between condition.

## Discussion

Implementation of a "Mid" (wet bathrobe), cooling maneuver during the break between two intermittent exercise periods elicited a three times greater Tre fall. This effect was carried on to the second exercise period where the rate of Tre rise was lower, thus preventing excessive hyperthermia [5].

## Conclusion

These results suggest an effective and practical mid-competition cooling maneuver.
